# Cost-utility and cost–benefit analysis of a multi-component intervention (NEXpro) for neck-related symptoms in Swiss office workers

**DOI:** 10.1186/s12889-024-21103-6

**Published:** 2025-01-15

**Authors:** Beatrice Brunner, Andrea Martina Aegerter, Venerina Johnston, Thomas Volken, Manja Deforth, Gisela Sjøgaard, Achim Elfering, Markus Melloh, the NEXpro Collaboration Group, Beatrice Brunner, Andrea Martina Aegerter, Venerina Johnston, Thomas Volken, Manja Deforth, Gisela Sjøgaard, Achim Elfering, Markus Melloh, Marco Barbero, Jon Cornwall, Yara Da Cruz Pereira, Oliver Distler, Julia Dratva, Holger Dressel, Tobias Egli, Markus J. Ernst, Irene Etzer-Hofer, Deborah Falla, Michelle Gisler, Michelle Haas, Sandro Klaus, Gina M. Kobelt, Kerstin Lüdtke, Hannu Luomajoki, Corinne Nicoletti, Seraina Niggli, Achim Nüssle, Salome Richard, Nadine Sax, Katja Schülke, Lukas Staub, Thomas Zweig

**Affiliations:** 1 ZHAW Zurich University of Applied Sciences, Winterthur Institute of Health Economics, Winterthur, Switzerland; 2 ZHAW Zurich University of Applied Sciences, School of Health Sciences, Institute of Public Health, Winterthur, Switzerland; 3 University of Southern Queensland, School of Health and Medical Sciences, Ipswich, Australia; 4 The University of Queensland, School of Health and Rehabilitation Sciences, Brisbane, Australia; 5 University of Zurich, Epidemiology, Biostatistics and Prevention Institute, Department of Biostatistics, Zurich, Switzerland; 6 University of Southern Denmark, Department of Sports Science and Clinical Biomechanics, Odense, Denmark; 7 University of Bern, Institute of Psychology, Bern, Switzerland; 8 Queensland University of Technology, School of Public Health and Social Work, Brisbane, QLD Australia

**Keywords:** Cost-utility, Neck pain, Workplace intervention, Health economic evaluation, Quality adjusted life years, Presenteeism, Randomized controlled trial

## Abstract

**Background:**

Neck pain is a significant public health issue, especially among office workers, with a prevalence ranging from 42 to 68%. This study aimed to evaluate the cost-utility and cost-benefit of a multi-component intervention targeting neck pain in the general population of office workers in Switzerland. The 12-week multi-component intervention consisted of neck exercises, health promotion information workshops, and workplace ergonomics sessions.

**Methods:**

The study was designed as a stepped-wedge cluster randomized controlled trial and assessed using an employer’s perspective. The main analysis focused on the immediate post-intervention period. Long-term effects were examined in a subsample at the 4, 8, and 12-month follow-ups. The intervention effects on costs and quality-adjusted life years (QALYs) were estimated using generalized linear mixed-effects models, controlling for confounding factors. Incremental cost-effectiveness ratios (ICERs) and cost-effectiveness acceptability curves were presented, along with calculations of the break-even point and the return on investment. Various sensitivity analyses were performed.

**Results:**

A total of 120 office workers participated in the trial, with 100 completing the intervention period and 94 completing the entire study. The main analysis included 392 observations. The intervention had a significant positive effect on QALYs and a nonsignificant effect on costs. The ICER was estimated at -25,325 per QALY gain, and the probability of the intervention being cost saving was estimated at 88%. The break-even point was reached one week after the end of the intervention.

**Conclusion:**

The multi-component intervention is likely to reduce company costs and simultaneously improve the quality of life of employees. However, the implementation of such interventions critically depends on evidence of their cost-effectiveness. As there is still a large research gap in this area, future studies are needed.

**Trial registration:**

ClinicalTrials.gov, NCT04169646. Registered 15 November 2019-Retrospectively registered.

**Trial protocol:**

Aegerter AM, Deforth M, Johnston V, Ernst MJ, Volken T, Luomajoki H, et al. On-site multi-component intervention to improve productivity and reduce the economic and personal burden of neck pain in Swiss office-workers (NEXpro): protocol for a cluster-randomized controlled trial. BMC Musculoskelet Disord. 2020;21(1):391. 10.1186/s12891-020-03388-x.

**Supplementary Information:**

The online version contains supplementary material available at 10.1186/s12889-024-21103-6.

## Introduction

Nonspecific neck pain is a serious public health problem in the general population of developed countries [1, 2]. With computer work being one of the risk factors, neck pain is particularly prevalent among office workers [3, 4]. The estimated annual prevalence of neck pain in office workers ranges from 42 to 68% [4, 5].

The impact of neck pain is significant. It has become one of the most common musculoskeletal disorders worldwide and is among the most frequently reported complaints in Western Europe [2, 6]. The costs associated with neck pain are correspondingly high and result from medical care utilization, reduced work productivity, and workers’ compensation claims. Thus, neck pain places not only a burden on the affected person, but also on society and employers [4].

Interventions at the workplace are becoming increasingly important for reducing the burden of neck pain. This is because companies are increasingly taking responsibility for the health of their employees, seeing the potential cost savings and productivity gains associated with a healthy workforce [4].

There are numerous studies evaluating the effectiveness of different workplace interventions for reducing neck pain. High-quality studies have demonstrated the positive effects of strengthening exercises and ergonomic changes in the workplace on neck pain severity among symptomatic office workers [4, 7] and on work productivity among a general population of office workers [8, 9]. However, studies on the cost-effectiveness of such programs are still scarce [10]. This is particularly surprising, as it is precisely this knowledge that is of central importance to companies that want to implement a workplace health program. Companies need to be able to compare the cost of the program to the company against the potential benefits to employee health and productivity to decide whether to implement a certain program.

To the best of our knowledge, the current knowledge about the cost-effectiveness of workplace interventions targeting neck pain is limited to the following three studies [11–13]. A Dutch study evaluated the cost-effectiveness of a work style (WS) intervention and a work style plus physical activity (WSPA) intervention in computer workers with neck and upper limb symptoms compared with usual care [11]. The WS intervention aimed at behavioural changes regarding body posture, workplace adjustment, breaks, and coping with risk factors for work stress. The WSPA interventions aimed to increase moderate- to high-intensity physical activity. Both intervention groups participated in six group meetings over a period of six months. The usual care group did not participate in the meetings but received the same reminders for breaks and exercises as the intervention group. The WS intervention did not prove to be more effective than usual care in improving recovery but was effective in reducing pain. The WS intervention was considered more cost-effective than usual care if a company was willing to pay approximately EUR 900 for a 1-point reduction in average pain. The WSPA intervention was not cost-effective [11]. Another Dutch study evaluated the cost-effectiveness of the repetitive strain injury (RSI) QuickScan intervention program for computer workers, which is already frequently used by companies [12]. The program included different interventions recommended based on a previously completed risk profile. It did not prove to be cost-effective, neither from a social nor an employer’s perspective [12]. A Swedish study evaluated the WorkUP trial and showed that a workplace dialogue with the employer is cost-effective from both a societal and a healthcare perspective for patients with acute and subacute neck and/or back pain who all received structured evidence-based physiotherapy [13].

To date, there is no study on the cost-effectiveness of a multi-component intervention. Multi-component interventions combine, e.g., exercises, health information, and ergonomic advice, and are considered to be more effective than single-component interventions, as this holistic approach addresses the different sources of pain [9]. Moreover, none of the previous studies considered neck pain-related presenteeism, i.e., reduced work productivity because of neck pain, to be an indirect cost. Finally, only the RSI QuickScan intervention study [12] included the general population of office workers, while the others focused exclusively on symptomatic individuals.

Our “Neck Exercise for Productivity NEXpro” study evaluates the cost-utility and cost-benefit of a multi-component intervention in the general population of office workers in Switzerland from an employer’s perspective. The time frame is immediately after the 12-week intervention for all participants and up to 12 months after the end of the intervention for a subsample of the participants. The effectiveness of the NEXpro intervention in terms of neck pain-related work productivity loss has already been demonstrated in an earlier paper [9].

## Methods

### Design

The cost-utility and cost-benefit analyses were conducted alongside the stepped-wedge cluster randomized controlled trial NEXpro, which took place between January 2020 and April 2021 among Swiss office workers [9]. The NEXpro trial (e.g., randomization, blinding) has been described in detail in two previous papers [9, 14]. Participants were randomized into three clusters, each consisting of five groups of eight participants. At four-month intervals, one cluster after the other received the intervention, while the remaining clusters served as a control group. This means that each participant eventually received the intervention; participants from cluster 1 between January and April 2020, participants from cluster 2 between August and December 2020, and participants from cluster 3 between January and April 2021. There was no cluster in the intervention period between April and August 2020 due to the time needed to adapt to the changing conditions for on-site interventions at the beginning of the Corona-19 pandemic. A detailed trial flow chart with adaptions made due to the Corona-19 pandemic can be found in an earlier paper [9].

The study was approved by the Ethics Committee of the Canton of Zurich, Switzerland (swissethics no. 2019–01678) and registered at clinicaltrials.gov (NCT04169646). The CONSORT 2010 Statement was used to guide the reporting of the trial [15].

### Study population

For the NEXpro trial [9], office workers were recruited at the end of 2019 from two governmentally funded Swiss organizations in the cantons of Aargau and Zurich [14]. Information about the study was disseminated via email and intranet announcements. Those interested in participating could register on a website and were screened for inclusion and exclusion criteria. For inclusion, office workers had to be between 18 and 65 years old, work at least 25 h per week predominantly in a sitting position (0.6 full-time equivalent), suffer from neck pain or be interested in the prevention of neck pain, understand written and spoken German, and provide written informed consent. Office workers were excluded if they suffered from severe neck pain (i.e., neck pain grade 4, [16]), planned an absence of more than four weeks during the intervention period, were pregnant, or were not allowed to perform neck exercises (e.g., on medical advice). A detailed list of all inclusion and exclusion criteria and a full description of the recruitment and randomization process can be found in the primary outcome paper [9].

### Recruitment

We contacted 1,333 employees (Aargau *N* = 540, Zurich *N* = 793), from which we screened the first 133 respondents based on a first-come, first-served basis for eligibility criteria. Of these, 7 office workers were excluded (prolonged absence from work *N* = 4, pregnancy *N* = 1, not sedentary office work *N* = 1, severe neck pain *N* = 1), and six declined to participate. Thus, a total of 120 office workers were included in the trial (i.e., our sample for baseline measurement), 107 participants started their assigned intervention period, 100 participants completed their respective intervention period, and 94 participants completed all follow-up measurements (i.e., our subsample for 4, 8, and 12-month post-intervention analysis) [9].

### Multi-component intervention

All participants received the same intervention during their allocated 3-month intervention period: neck exercises, health-promotion information workshops, and a workstation ergonomics intervention [9]. Furthermore, they were instructed to continue their regular work and leisure activities as usual. All interventions were supposed to be on-site at the office. Due to the Corona-19 pandemic, the interventions were partially delivered online from March 2020 onwards [9].

Neck exercises were performed three times a week for at least 20 min; one session was held in a group and with a physiotherapist during usual office hours, and the other two sessions were completed individually outside business hours. Participants were given access to an application for their digital devices, where they could choose from a standard set of 16 neck exercises, including ten strengthening and six non-strengthening exercises. The training material (e.g., elastic resistance bands) was provided at no cost.

Twelve weekly health-promotion information group workshops of 45 min each were held by healthcare professionals during work hours. Topics such as work stress, mental health, resilience, and mindfulness were discussed.

Workstation ergonomics intervention was conducted once and individually with a physiotherapist or movement scientist for 30 min during work hours. The workstation was set up according to best-practice ergonomic guidelines, using only the existing infrastructure (cost-neutral). Adjustments were made to the chair, desk, and monitor as needed. A detailed description of the multi-component intervention is provided in an earlier paper [9].

### Outcome measures

The data were collected using online questionnaires at baseline (January 2020) and at four follow-up timepoints at four-month intervals (follow-up 1 in April 2020, follow-up 2 in August 2020, follow-up 3 in December 2020, and follow-up 4 in April 2021). Measurements were made at the same timepoint for all participants, regardless of whether they were in the control or intervention group.

We measured health-related quality of life using the German version of the EQ-5D-5L questionnaire [17]. Quality-adjusted life years (QALYs) were calculated based on the EuroQol Group’s German value set [18]. One QALY is equivalent to one year in perfect health.

We also collected data on the intensity of neck pain (Numerical Rating Scale NRS from 0 = no pain to 10 = maximum pain), neck disability (Neck Disability Index NDI from 0% = no disability to 100% = maximum disability), and neck pain frequency (number of days in the previous month with neck pain, scale between 0 and 28 days) [14, 19, 20].

### Cost data

The cost-utility and cost–benefit analyses are conducted from an employer’s perspective. Therefore, all costs related to the intervention that are relevant to the employer are included. These are on the one hand the costs of the intervention itself, and on the other hand the costs of neck pain-related productivity losses. The costs of measurement and data collection (e.g., completing the questionnaire during work time) were not considered. No costs occurred for participants who were in the control period.

The costs of the intervention were calculated for each participant by summing the costs from an employer perspective and the program costs. The costs from the employer's perspective included the costs incurred by allowing participants to attend the intervention during work time. These costs were indicated separately for the three intervention components (exercise, workshop, ergonomics), as well as for the time participants travelled between their office and the intervention room (e.g., five minutes). The program costs consisted of the health care professional’s time to prepare and deliver the intervention, the material used (e.g., elastic rubber band, access to smartphone application), and their travel costs to the organizations (i.e., train ticket, time). Due to the Corona-19 pandemic, two different cost calculations were made: an on-site calculation for the main analysis, and an online calculation for a sensitivity analysis.

The costs of neck pain-related productivity losses included costs due to absenteeism and presenteeism. Absenteeism corresponds to the time absent from paid work and presenteeism corresponds to reduced productivity while working. Both refer exclusively to impairments due to neck pain and were assessed using the Work Productivity and Activity Impairment Questionnaire for Specific Health Problem (WPAI SHP, German version 2.0) [21]. The WPAI questionnaire includes five questions (Q1 to Q5) with a recall time frame of one week: Q1 = currently employed; Q2 = hours missed due to neck pain; Q3 = hours missed due to other reasons (e.g., vacation); Q4 = hours actually worked; and Q5 = degree to which neck pain affected work productivity (on an NRS ranging from 0 = not at all to 10 = maximum) [21]. We followed the coding and scoring rules of the developers to obtain the neck pain-related work productivity losses expressed as a percentage of working time: Q2/(Q2 + Q4) + (1-Q2)/(Q2 + Q4) × Q5/10 [21]. We assumed that neck pain-related productivity losses in the week prior to each follow-up measurement were representative of the four-month period between the current and previous measurements [9]. The monetary value of the neck pain-related productivity losses is calculated based on the human capital approach using individual earnings (in Swiss Francs, CHF). In the main analysis, an elasticity between work time and work productivity of 1 is used, which means that an impairment of 10% of the working time also leads to a productivity loss of 10%. Lower elasticities are used as part of the sensitivity analysis.

### Sample size

The sample size calculation was based on the primary outcome of the NEXpro project –, i.e., neck pain-related work productivity loss – and resulted in 120 participants over four measurement time points, yielding 480 observations (baseline work productivity of 90%, assumed intervention-related work productivity increase of 5%, Type I Error alpha = 0.05, Type II Error beta = 20%, Power = 80% [9]). However, due to adjustments from the Corona-19 pandemic, a fifth measurement time point was included, resulting in 600 observations.

The number of observations used in this article had to be slightly limited due to the time-dependent nature of the cost-utility analysis and our stepped-wedge design. The main analysis included a total of 392 observations (120 treated participants), with 295 observations in the control period and 97 observations immediately after the end of their assigned intervention period. For the additional analysis of longer-term effects, only subsample data are available. The number of observations in the subsamples of the 4-, 8-, and 12-month post-intervention analyses were 357 (control *N* = 295, intervention *N* = 62; 80 treated participants), 327 (control *N* = 295, intervention *N* = 32; 40 treated participants), and 326 (control *N* = 295, intervention *N* = 31; 40 treated participants), respectively (Additional file, Table A1). Since these subsamples are significantly underpowered, the longer-term effects should be interpreted with caution.

### Time horizon

A trial flow chart is provided in an earlier paper [9]. In the main analysis, we estimate the program effects immediately after at the 3-months intervention period, as this was the only post-intervention period observed for all 120 treated participants. Specifically, the first measurements immediately after the end of the intervention period were at follow-up 1 for cluster 1, at follow-up 3 for cluster 2, and at follow-up 4 for cluster 3 (Additional file, Table A1).

In an additional analysis, we estimate the longer-term effects at 4-, 8- and 12-months post-intervention, using the subsamples of participants from clusters 1 and 2 (Additional file, Table A1), with the sample sizes explained in the previous subsection.

### Statistical model

The intervention effect on costs was estimated by fitting a generalized linear model of the Gaussian family with log-link. A beta regression model was used to estimate the effect of the intervention on QALYs. In either case, robust standard errors are estimated, and the model includes a dummy variable for group allocation (intervention, control), fixed effects for the cluster (cluster 1, 2 or 3), fixed effects for the measurement time point (baseline, follow-up 1, 2, 3, or 4) and a random intercept to account for repeated measurements on the same participants. Furthermore, the models control for potential confounding effects by including age, gender, education level, civil status, nationality, employer, workload percentage, work role, and work stress. The latter was assessed using the Job-Stress-Index (JSI), which ranges from 0 to 100 and enables the classification into a favourable range (resources > stressors, if JSI < 45.88), a sensitive range (resources = stressors, if 45.88 ≤ JSI < 54.12) and a critical range (resources < stressors, if JSI > 54.22) [22]. In selecting the potential confounders, we followed the model of our primary outcome paper [9]. Finally, average marginal effects were derived from the models to represent the estimated intervention effects on costs and QALYs.

### Cost-utility analysis

The incremental cost-effectiveness ratio (ICER) was calculated by dividing the average marginal effect on costs by the average marginal effect on QALYs and was graphically presented on a cost-effectiveness plane (CEP). The uncertainty of the CEP is represented by confidence ellipses covering 50%, 75% and 95% of the joint density of the cost and QALY effects [23, 24]. The cost-effectiveness acceptability curve (CEAC) is calculated and graphically presented, showing the probability of the intervention being cost-saving and cost-effective at a specific ceiling ratio, respectively [23]. The time horizon for both ICER and CEAC is 3 months, i.e. immediately at the end of the intervention period.

### Cost-benefit analysis

We calculated the break-even point (in days) and the estimated savings one year after the end of the intervention, based on the estimation results obtained immediately after the 3-month intervention period using the full sample (main analysis), as well as considering long-term effects at 4-, 8- and 12-months post-intervention using subsamples (additional analysis).

### Sensitivity analysis

To assess the robustness of the main results, three sensitivity analyses were conducted.

The first sensitivity analysis consisted of a scenario in which the intervention was conducted completely online. The original plan of the NEXpro project was to conduct a multi-component intervention on-site at the office. However, due to the Corona-19 pandemic, it had to be changed to a hybrid setting, i.e., on-site and online. As the costs for an online intervention are lower than those for an on-site intervention – and the online intervention is likely to become more important in the future – this first sensitivity analysis was performed.

The second sensitivity analysis relates to the elasticity between working time and work productivity, as there is evidence suggesting that lost work due to absenteeism and presenteeism is partly compensated without incurring productivity costs [25, 26]. This would imply an elasticity below 1, whereas we defined an elasticity of 1 for our main analysis. The findings of a study comparing the relationship between objectively measured work productivity and employee-reported work productivity using a questionnaire similar to the WPAI suggest an elasticity of 0.4 to 0.5 [25]. The generalizability of their results is questionable, however, as their study was carried out in a single work setting (among American call centre workers), and they were only able to measure work quantity and not quality [25]. A Dutch study estimated the same for a more general setting and suggested an elasticity of 0.8 [26]. Based on these results we conducted two sub-sensitivity analyses with elasticity values of 0.5 and 0.8, indicating that an impairment of 10% due to neck pain will lead to production losses of 5% and 8%, respectively. This also implies that the remaining 5% or 2% will be compensated by his or her colleagues or by the affected worker at a later stage.

The third sensitivity analysis relates to the estimation method of the cost-utility results. Instead of estimating the intervention effects on costs and QALYs separately, we estimated them simultaneously using a Seemingly Unrelated Regression (SUR) model [27]. The SUR model is often applied in health economic analyses because it accounts for the correlation of error terms of the two equations (the correlation is -0.17 in our data) [28]. The disadvantage of the SUR model, however, is that it assumes a bivariate normal (or lognormal) distribution of costs and QALYs [27]. For QALYs, this assumption is clearly violated in our case (Additional file, Figure A1).

## Results

### Descriptive statistics at baseline

Table 1 shows the participants’ characteristics at baseline. The average participant was female (71.7%), 43.7 years old (SD 9.8), Swiss (79.2%), in a relationship (married: 40.0%, not married: 44.2%), had a tertiary level education (74.2%), and had a JSI of 47.6 (SD 5.0). Furthermore, the average participant was employed by the company in Zurich (53.3%), worked full-time (79.2%), had been employed there for approximately 3 to 5 years (27.5%), held no leadership position (63.3%), and earned CHF 7,679 per month (SD 2,818).

**Table 1:** Participant characteristics at baseline, *N* = 120

Characteristics	Categories	Mean (Categories: in %)	SD
Age (years)		43.7	9.8
Gender	female	71.7	
	male	28.3	
Education	tertiary level	74.2	
	non-tertiary level	25.8	
Marital status	in a relationship, not married	44.2	
	in a relationship, married	40.0	
	single	15.8	
Nationality	Swiss	79.2	
	Non-Swiss	20.8	
Job-Stress-Index (0–100)		47.6	5.0
Job-Stress-Index (categories)	resources = stressors	45.0	
	more resources	41.7	
	more stressors	13.3	
Employer	company in Zurich	53.3	
	company in Aargau	46.7	
Workload	100%	40.0	
	80–99%	39.2	
	< 80%	20.8	
Leadership responsibility	No	63.3	
	Yes	36.7	
Tenure	< 2 years	23.3	
	3–5 years	27.5	
	6–10 years	24.2	
	> 10 years	25.0	
Income per month (CHF)		7679	2818

The duration of the intervention period for each participant was 12 weeks (i.e., 3 months), with an average of 31.2 training sessions of neck exercises completed (ranging from 0 to 93 training sessions; equal to 2.6 training sessions per week), 8.2 attendances of group health promotion workshops (ranging from 0 to 12 attendances), and 97.2% of participants (*N* = 104/107) participated in the individual workplace ergonomics session [9].

Table 2 presents the baseline values for QALYs and neck pain-related outcomes (upper panel) as well as neck pain-related work productivity losses (lower panel). This table also shows the proportion of participants reporting the best possible value for each measure (last column, “value share”). The average QALYs were 0.92 for all participants and at 0.89 when excluding participants who reported the best possible QALY of 1 (24.2% reported the best possible QALY of 1).

**Table 2:** Health measures and neck pain-related work productivity loss at baseline

	Mean	Median	SD	Best possible value (share)
Primary outcome measure of this analysis				
* QALY [0–1]*	0.92	0.94	0.09	24.2%
Neck pain-related outcome measures				
*Pain Intensity [0–10]*	2.4	2.0	2.0	21.7%
*Pain Frequency [0–28]*	6.8	4.0	8.0	20.8%
*Disability [0–100]*	11.8	12.0	9.9	21.7%
Neck pain-related work productivity loss (in % of working time)				
*Absenteeism [0–100]*	1.2	0.0	9.2	92.5%
* Presenteeism [0–100]*	10.8	0.0	16.9	51.7%
*Total productivity loss [0–100]*	12.0	0.0	19.4	50.8%

Key: *N* = 120. Note: Effectiveness of the intervention on neck pain-related outcome measures will be published in a separate paper

The average neck pain intensity was 2.4/10 on the NRS for all participants and 3.0/10 on the NRS when excluding participants who reported the best possible NRS score of 0 (21.7% reported the best possible value of NRS 0/10, Table 2). The average frequency of neck pain was 6.8 days/month for all participants and 8.6 days/month when excluding participants who reported the best possible frequency of 0 days/month (20.8% reported the best possible value of 0 days). The average neck disability was 11.8% for all participants and 15.1% when excluding participants who reported the best possible neck disability of 0% (21.7% reported the best possible value of 0%). This is important for the subsequent interpretation of the results as the intervention cannot lead to a further increase in QALYs for nearly one fifth of the participants.

The average total neck pain-related work productivity loss was 12.0% of working time for all participants and 24.4% of working time when excluding participants who reported the best possible value of 0% (50.8% reported the best possible value of 0%). Table 2 also shows that the majority of productivity losses are due to presenteeism (10.8% of 12.0%) and that 92.5% of all participants achieved the best possible value of 0% of working time for absenteeism.

Table 3 shows descriptive statistics of the intervention costs (upper panel) and the monthly neck pain-related work productivity losses (in CHF) at baseline (lower panel). The intervention costs were CHF 770.5 (SD 220.0) per person and 3-month on-site intervention, with the largest share being employer costs of CHF 475.1 (SD 189.3), i.e., the working time spent by participants on the intervention (Table 3). Almost two-thirds of the employer costs were for workshop participation (mean CHF 297.9, SD 125.4). The program costs were mainly due to the travel costs of the health professionals (e.g., time, train ticket) amounting to CHF 127.2 (SD 103.5) per participant. The online cost calculation was less expensive because there were fewer material costs (e.g., no printing costs) and no travel costs. The intervention costs for an online scenario were CHF 613.3 (SD 186.2) per person and 3-month intervention (sensitivity analysis).

**Table 3:** Neck pain-related productivity losses and intervention costs, per person

	**On-site**			**Online**		
	Mean	SD	Median	Mean	SD	Median
**Intervention costs, *N* = 120**	**770.5**	**220.0**	**777.9**	**613.3**	**186.2**	**609.4**
Employer costs^a^	475.1	189.3	472.5	448.3	183.5	447.4
* Workstation ergonomics*	*23.5*	*7.6*	*21.7*	*23.5*	*7.6*	*21.7*
* Health-promotion information workshop*	*297.9*	*125.4*	*304.2*	*297.9*	*125.4*	*304.2*
* Neck exercises*	*126.9*	*56.1*	*123.5*	*126.9*	*56.1*	*123.5*
* Travel costs*	*26.8*	*10.5*	*25.1*	*0.0*	*0.0*	*0.0*
Program costs^b^	295.3	109.8	248.0	164.9	22.1	158.7
* Workstation ergonomics*	*21.2*	*0.0*	*21.4*	*21.1*	*0.0*	*21.1*
* Health-promotion information workshop*	*46.6*	*10.4*	*47.8*	*43.5*	*10.1*	*44.7*
* Neck exercises*	*100.3*	*12.7*	*97.8*	*100.3*	*12.7*	*97.8*
* Travel costs*	*127.2*	*103.5*	*43.3*	*0.0*	*0.0*	*0.0*
Did not finish intervention period *N* = 20	*-*	*-*	*-*	*-*	*-*	*-*
Missing *N* = 3	*-*	*-*	*-*	*-*	*-*	*-*
**Monthly neck pain-related work productivity loss at baseline, *N* = 120**						
Absenteeism	44.6	254.2	0	44.6	254.2	0
Presenteeism	771.3	1240.2	0	771.3	1240.2	0
Total productivity loss	815.9	1305.9	0	815.9	1305.9	0

Key: a) Working time spent by participants on the intervention, b) Working time spent by the health-care professionals for preparation and implementation of the intervention as well as material (e.g., training material, train ticket). Note: For the main analysis, an elasticity between work time and work productivity of 1 is assumed. The average intervention cost per person is CHF 613.3 if the intervention is conducted online (sensitivity analysis i.). The average productivity loss per person is CHF 652.7 with an elasticity of 0.8 (sensitivity analysis 2.i), and CHF 408.3 with an elasticity of 0.5 (sensitivity analysis 2.ii)

The monthly neck pain-related work productivity loss was CHF 815.9 (SD 1305.9), which with a share of CHF 771.3 (SD 1240.2) mainly consisted of costs due to presenteeism (Table 3, lower panel). As expected, these values decrease with an elasticity of 0.8 to CHF 652.7, and an elasticity of 0.5 to CHF 408.3.

### Effects on QALYs and costs

The estimated marginal effects of the intervention are shown in Table 4, panel A. In terms of effectiveness, a significant positive effect was found. On average, QALYs increased by 0.028 [95% CI: 0.008 to 0.049]. In terms of costs, we found an insignificant average marginal effect of CHF -720 [95% CI: -1906 to 466]. Thus, the reduction in neck pain-related work productivity losses more than offset the intervention costs on average.

**Table 4:** Estimated marginal effects, cost-effectiveness and cost-benefit

	Marginal effects		Cost-utility		Cost-benefit	
	Cost difference (CHF)	Effect difference (QALY)	ICER	Probability of being cost-saving	Break-even (days)^b^	1 year post-intervention return^a^
**A. Main analysis**	-720	0.028***	-25325	0.88	6.4	3.67
[95% CI]	[-1906; 466]	[0.008; 0.049]				
**B. Sensitivity analyses**^c^						
1. Conducted online	-813	0.028***	-28600	0.91	-23.6	5.24
[95% CI]	[-1990; 364]	[0.008; 0.049]				
2.i. Elasticity of 0.8	-497	0.028***	-17483	0.85	50.2	2.58
[95% CI]	[-1454;460]	[0.008; 0.049]				
2.ii. Elasticity of 0.5	-147	0.028***	-5193	0.67	386.8	0.76
[95% CI]	[-775;480]	[0.008; 0.049]				
3. SUR	-420	0.037**	-11312	0.72	76.1	2.18
[95% CI]	[-1825; 983]	[0.007; 0.067]				

Key: *N* = 392. Significance levels * 10%, ** 5%, *** 1%. a) The return after one year post-intervention is shown as return for each CHF invested. b) The break-even-point is shown in days after the end of the intervention. c) Sensitivity analysis: 1. The intervention was conducted online only; 2.i. and 2.ii The elasticity of working time to work productivity is assumed to be 0.8 and 0.5, respectively; 3. A seemingly unrelated regression (SUR) is used for estimation. Note: For the marginal effects and the cost-utility results, the time horizon is 3 months, concluding with the end of the program. The models were adjusted for participant characteristics (Additional file, Table A2). The cost-benefit calculations are based on subsamples (Additional file, Table A1) and the time horizon is 12 months post-intervention

**Fig. 1:**
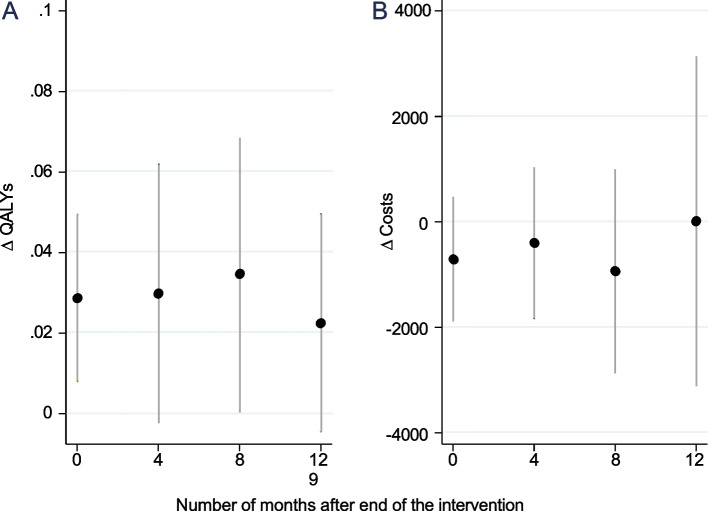
PPersistence of effects after the end of the intervention. **Panel A** shows the change in QALYs at 0, 4, 8, and 12 months after the end of the intervention. **Panel B** shows the change in costs at 0, 4, 8, and 12 months after the end of the intervention. Key: *N* = 392, 357, 327, 326 at post-intervention month 0, 4, 8 and 12. The points represent the average marginal effects of the intervention at different post intervention follow-up time periods, the bars represent the 95% confidence intervals

In addition to the immediate effects at the end of the intervention, our stepped-wedge design allows us to estimate long-term effects for subsamples of our population (Fig. 1). More specifically, we estimated the impact at 4, 8 and 12 months after the end of the intervention. Both the effects on QALYs and costs appear quite stable over the whole observation period. However, as the sample sizes decrease over time, the estimates should be interpreted with caution – an issue further discussed in the limitations section. Nevertheless, the results provide an indication of the potential persistence of the intervention effects.

### Cost-utility

**Fig. 2:**
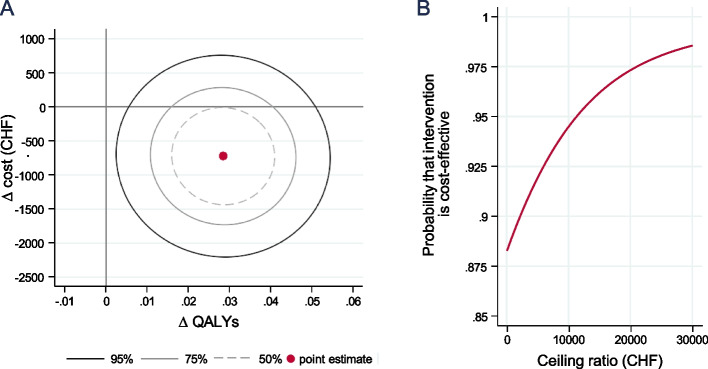
CEP and CEAC. **Panel A** shows the cost-effectiveness plane (CEP) with the point estimate representing the average marginal effect on QALYs and costs (= ICER). Uncertainty is represented by the confidence ellipses. **Panel B** shows the corresponding cost-effectiveness acceptability curve (CEAC) Key: *N* = 392

The significant difference in QALYs of 0.028 in favour of the intervention, and the non-significant difference in the costs of CHF -720, resulted in an ICER of CHF -25,325 per QALY from an employer’s perspective (Table 4). In other words, the intervention was cost saving for the average participant. This can also be seen in the cost-effectiveness plane (CEP; Fig. 2A). The majority of the joint density distributions of the estimated intervention effects lies in the lower right quadrant, indicating that the intervention led to an increase in QALYs and a decrease in costs for most of the participants. In addition, the cost-effectiveness acceptability curve (CEAC) shows that the probability of the intervention being cost saving is estimated at 88% (Fig. 2 B and Table 4, column 7). It also shows that the intervention has a 95% probability of being cost-effective at a threshold of CHF 11,000 per QALY gained, or put differently, at a willingness of pay of CHF 308 per participant (= 0.028* CHF 11,000).

### Cost-benefit

Tables 3 and 4 show that by the end of the 3-month intervention period, the costs per employee (CHF 770.5 on average) slightly exceed the financial returns (CHF 720 per employee). This translates to a savings rate of CHF 0.93 for each Swiss Franc invested. Furthermore, the findings in Fig. 1 suggest that productivity gains continue beyond the program’s end. Based on Fig. 1, we make the following assumption needed to calculate the break-even point and estimated savings one year after the program concludes: productivity gains are assumed to be stable and uniformly distributed throughout the year following the intervention. Under this assumption, two key findings stand out:

Firstly, the program reaches its break-even point on average one week (6.4 days) after the intervention ends, indicating that the financial benefits from increased employee productivity offset the program costs within just over six days.

Secondly, within one-year post-intervention, the program costs remain at CHF 770.5 per employee, while expected savings from productivity gains are estimated at CHF 2,829.5 per employee. This translates to a projected return of CHF 3.67 for each CHF invested, driven by improved employee productivity due to reduced neck pain.

### Sensitivity analysis

Table 4, panel B, shows how our main results change as a result of the sensitivity analyses. First and foremost, it is important to highlight that, on average, the intervention proves to be both effective and cost-saving across all sensitivity analyses, with variations only in the likelihood of it being deemed cost-saving. The highest probability is achieved if the intervention would have been conducted entirely online (sensitivity analysis 1). In this scenario, the intervention is estimated to be cost saving with a 91% probability, and the average break-even point occurs 24 days before the program concludes. This outcome is not surprising, as an online format reduces costs, such as travel expenses. The probabilities are lower than in the main analysis if we assume an elasticity between hours worked and work productivity of less than 1 (sensitivity analysis 2). In fact, there is a positive correlation between the elasticity level and both the likelihood of cost savings and the size of the return. This is explained by the fact that as the elasticity decreases, the positive effects of the intervention on productivity losses turn into positive productivity effects to an increasingly lesser extent. The third and final sensitivity analysis consisted of simultaneously estimating the impact on QALYs and costs using SUR. Compared to our main analysis, SUR had a larger marginal effect on QALYs (0.037 compared to 0.028) and a lower, but also insignificant, marginal effect on costs (CHF -420 compared to -720). The probability of the intervention being cost saving when estimated with SUR was 72% and the break-even point is reached 76 days after the end of the intervention.

## Discussion

In the present study, we evaluated the cost-utility and cost-benefit of the multi-component intervention NEXpro, which consists of neck exercises, health-promotion information workshops and ergonomic workstation adjustments over a period of three months in the general population of office workers in Switzerland. The intervention significantly reduced neck pain-related work productivity losses (as shown in an earlier paper [9]) and increased QALYs immediately after the end of the intervention. The results based on subsamples also indicate a longer-term effect. Additionally, the intervention proved to be economically beneficial from the employer’s perspective. Within just over six days post-intervention, the program reached its break-even point, considering only productivity gains from reduced absenteeism and presenteeism. Further savings may arise from reduced turnover and enhanced employee morale. Thus, implementing such interventions has the potential to lower company costs while improving employees' quality of life – a true win-win situation.

When comparing our effectiveness results with studies that only include symptomatic individuals, as most studies have to date, it is important to note that asymptomatic individuals dilute the effects of the intervention to some extent, as these people may already have had maximum QALY values at baseline. Consequently, the intervention cannot lead to any improvement in their values. While we cannot derive the true effect for symptomatic individuals from our results, we have attempted to narrow it down. Assuming that asymptomatic individuals are those with maximum QALYs, the effect would be 0.037 (= 0.028/(1–0.242)). Few cost-effectiveness studies exist that could be used to compare our effectiveness results. They were all conducted in the healthcare setting and not in the workplace. A recent study among individuals (18 to 70 years) with subacute or persistent neck pain revealed QALY gains from exercises targeting the neck, chest, scapula and jaw of 0.03 after 3 months, 0.039 after 6 months and 0.04 after 12 months [29]. A second study among working age patients at risk of sick leave due to acute neck or back pain reported QALY gains of 0.033 after 12 months of evidence-based physiotherapy [13]. Our combined intervention achieved QALY gains of 0.04 in a shorter time compared to these studies, which only observed QALY gains of almost 0.04 after 12 months of intervention.

Various sensitivity analyses were performed, all of which confirmed the robustness of our results. For our first sensitivity analysis, we used different values for the elasticity between hours worked and work productivity since there is some evidence suggesting values below 1 due to compensation mechanisms [25, 26]. We found that the probability of the intervention being a cost saving decreases with decreasing elasticity. With an elasticity of 1, we found a probability of 88%, and with an elasticity of 0.5, we found a probability of 67%. This positive correlation can be explained by the fact that elasticity acts as a weight for productivity gains. The smaller the elasticity is, the smaller the weight of the beneficial productivity effects, and the less likely cost savings are from the intervention. While we do not know the true elasticity, it probably depends on the type of work. Work with simpler content is much more likely to be taken on by colleagues than demanding work that requires very specific knowledge.

Our second sensitivity analysis concerns the way the intervention was carried out. The online version (sensitivity analysis) proved to be even more cost-effective than the on-site version (main analysis) from the employers’ perspective (91% vs. 88% probability of being cost saving). This is due to the lower program costs of the online version compared to the on-site version (i.e., no travel costs). In addition to the travel costs saved with the online version, program costs could be almost completely eliminated in future implementations of the intervention, as they were mainly due to the time spent by healthcare professionals preparing for the intervention (i.e., producing neck exercise videos for the smartphone application or creating workshop materials). For this reason, we tested a fully online intervention with office workers at the University of Bern in a follow-up project of NEXpro (WeMoveVirtual) [30]. In the next step, cross-validation could be carried out based on these data. However, it is important for the success of the intervention that office workers are able to participate during work hours, which is why no substantial change in employer costs can be expected, even if the intervention is implemented online.

To the authors’ knowledge, this is the first cost-utility and cost-benefit evaluation of a multi-component RCT at the workplace aiming to prevent or reduce neck pain in office workers. A strength of the study is that the cost-utility analysis was conducted alongside the RCT, therefore limiting the risk of bias. Another strength is that the analysis evaluates the cost-utility and cost-benefit of the entire multi-component intervention instead of comparing single interventions against each other. In this way, companies can directly see how likely the intervention is to be worthwhile from a financial point of view. Another strength from an ethical point of view is that all participants received the intervention regardless of their group allocation as we also collected control group data.

This study also has several limitations. First, the NEXpro RCT was slightly underpowered to detect differences in QALYs and significantly underpowered to detect cost differences in the full sample (i.e., immediately after the intervention). The power issues were even more pronounced in the subsamples used to assess the longer-term effects, with sample sizes decreasing further as the post-intervention period lengthened (N treated = 62, 32, and 31 at 4, 8, and 12 months after the intervention, respectively; Additional File 1, Table A1). As a result, the estimates become increasingly uncertain and should be interpreted with appropriate caution. This is because the power calculation was undertaken for our primary outcome paper [9] based on productivity effects estimated using inter-temporal variation. In particular, for cost effects and all longer-term effects, a larger sample size would have been required to obtain robust results. Therefore, it is especially important to interpret the results on the persistence of the effects, and particularly the cost–benefit calculations, with caution. The latter are based on the average marginal effect of costs, which is not significant, as well as on assumptions derived from the estimates of the long-term effects.

Second, the NEXpro trial was performed in the Swiss context and in only two companies. Due to personnel, administrative, and financial constraints, we were unable to include companies from different types of industries, which affects the generalizability of our results. When using interventions in different settings, the population characteristics, as well as cultural, social, and political differences, need to be taken into consideration.

Third, we would like to highlight that the Corona-19 pandemic occurred during the study period. This raises the question of whether the pandemic might have influenced the study results (e.g., through changes in working conditions) or introduced a systematic bias. We accounted for this by including fixed effects for clusters and measurement time points (follow-ups) and by calculating the within- and between-cluster variability. We found no indication that the Corona-19 pandemic had a systematic effect on our results. Due to limited degrees of freedom, further calculations (e.g., treatment effects for different subsamples) were not possible.

Economic evaluations of effective interventions are essential for policymakers to make evidence-based, informed decisions. They can also increase the likelihood of such interventions being implemented at all. Since there is still a large research gap in this area, future studies evaluating the cost-effectiveness of workplace interventions targeting the neck and upper limb are needed.

## Supplementary Information

Supplementary Material 1.

## Data Availability

The data, code, and material that support the findings of this study are available from the corresponding author upon request.

## Abbreviations

CEAC: Cost-effectiveness acceptability curve
CEP: Cost-effectiveness plane
CHF: Swiss Francs
CI: Confidence interval
EUR: Euro
ICER: Incremental cost-effectiveness ratio
JSI: Job-Stress-Index
NEXpro: Neck Exercise for Productivity
NDI: Neck Disability Index
NRS: Numerical Rating Scale
RCT: Randomized controlled trial
RSI: Repetitive strain injury
SD: Standard deviation
SUR: Seemingly Unrelated Regression
QALYs: Quality-adjusted life years
WPAI: Work Productivity and Activity Impairment Questionnaire
WS: Work style
WSPA: Work style plus physical activity
ZHAW: Zurich University of Applied Sciences
