# Supplementary material for: Cost-utility and cost–benefit analysis of a multi-component intervention (NEXpro) for neck-related symptoms in Swiss office workers
Source: BMC Public Health. 2025 Jan 15;25:160. doi: 10.1186/s12889-024-21103-6 (PMC11734223; doi:10.1186/s12889-024-21103-6)
Supplement: Supplementary file 1 — Supplementary Material 1. [file 12889_2024_21103_MOESM1_ESM.pdf]

Cost-Utility and Cost-Benefit Analysis of a Multi-component Intervention (NEXpro) for Neck-related Symptoms in Swiss Office Workers

Additional file 1

Table A1: Number of observations split for each measurement time point.

	Number of observations included in main analysis *(missing, dropout)*	Control period *(missing, dropout*	Intervention period, time point after the end of the intervention *(missing, dropout)*			
			Immediately	4-months	8-months	12-months
**Total number of observations** *(missing, dropout)*	392 *(48)*	295[A, B,C] *(25)*	97 *(23)*	62[A] *(18)*	32[B] *(8)*	31[C] *(9)*
Baseline *(missing, dropout)*	120 *(0)*	120 *(0)*	0 *(0)*	0 *(0)*	0 *(0)*	0 *(0)*
Follow-up 1 *(missing, dropout)*	111 *(9)*	75 *(5)*	36 *(4)*	0 *(0)*	0 *(0)*	0 *(0)*
Follow-up 2[D] *(missing, dropout)*	69 *(11)*	69 *(11)*	0 *(0)*	32 *(8)*	0 *(0)*	0 *(0)*
Follow-up 3 *(missing, dropout)*	63 *(17)*	31 *(9)*	32 *(8)*	0 *(0)*	32 *(8)*	0 *(0)*
Follow-up 4 *(missing, dropout)*	29 *(11)*	0 *(0)*	29 *(11)*	30 (10)	0 *(0)*	31 *(9)*

Key: A: Number of observations included in 4-months post-intervention analysis (N=357, missing/dropout=43) B: Number of observations included in 8-months post-intervention analysis (N=327, missing/dropout=33) C: Number of observations included in 12-months post-intervention analysis (N=326, missing/dropout=34). D: No cluster changed in the intervention period due to the Corona-19 pandemic.

Table A2: Estimated effects on costs and QALYs after 3 months, detailed results.

	Cost (CHF)	QALY
Average marginal treatment effect	-720	0.028***
	(605)	(0.010)
Average treatment effect	-0.337	0.436*
	(0.308)	(0.176)
Measurement time point: follow-up 1 (April 2020)	0.165	0.184
	(0.207)	(0.209)
Measurement time point: follow-up 2 (August 2020)	0.047	-0.229
	(0.173)	(0.248)
Measurement time point: follow-up 3 (November 2020)	-0.282	0.269
	(0.331)	(0.280)
Measurement time point: follow-up 4 (April 2021)	0.532	-0.186
	(0.397)	(0.372)
Cluster 2 (ref. cluster 1)	-0.334	-0.026
	(0.263)	(0.284)
Cluster 3 (ref. cluster 1)	-0.215	0.394
	(0.260)	(0.277)
Age	−0.034***	-0.012
	(0.011)	(0.010)
Male (ref. Female)	-0.464*	-0.127
	(0.228)	(0.205)
Tertiary education (ref. non teriary)	-0.077	-0.088
	(0.280)	(0.229)
In relationship (ref. married)	0.498*	0.393
	(0.216)	(0.209)
Not in relationship (ref. married)	0.615	0.401
	(0.323)	(0.268)
Non-Swiss (ref. Swiss)	0.423	0.216
	(0.222)	(0.265)
Region Aarau (ref. Region Zurich)	0.096	-0.257
	(0.259)	(0.248)
Employed 90-99% (ref. 100%)	-0.124	-0.017
	(0.257)	(0.273)
Employed 80-89% (ref. 100%)	-0.184	-0.176
	(0.237)	(0.227)
Employed < 80% (ref. 100%)	-0.006	0.054
	(0.243)	(0.269)
Leadership function (ref. none)	0.828***	-0.116
	(0.235)	(0.206)
Job-stress-index	0.031*	0.046*
	(0.012)	(0.018)
Mean dependent variable	2546	0.925
SD dependent variable	3890	0.082
Number of observations	392	392
p-value (F-statistic)	0.000	0.000
Between-Cluster-Variability	0.579	0.000011[a]
Within-Cluster-Variability	6426895	0.006738[a]

Key: Detailed estimation results (coefficients and standard errors in parentheses) of the marginal effects are presented in Table 4, Panel A. Costs and QALYs were modelled using a GLME Log-Link and a Beta Regression Model, respectively. Time horizon is 3 months. Significance levels * 5%, ** 1%, *** 0.1%. [a] calculated manually. Note that the fixed effects for the measurement time points (follow-up 1 to 4) capture potential changes in treatment conditions during the Corona-19 pandemic.

Figure A1: Histogram of costs and QALYs

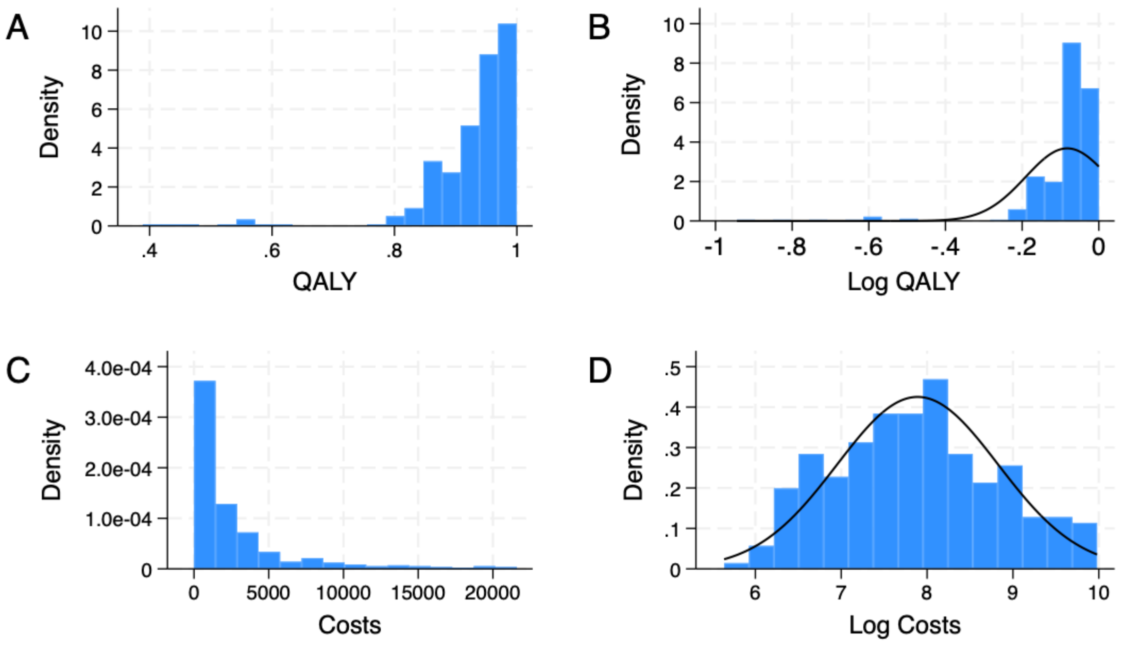

Key: Panel A and C show the density distribution of QALYs and costs, both measured at the end of the intervention (i.e. after 3 months). Panel B and D show the density distribution of the log QALYs and log costs, accordingly. Costs are measured in Swiss Francs (CHF).
